# Rab GTPase regulation of bacteria and protozoa phagocytosis occurs through the modulation of phagocytic receptor surface expression

**DOI:** 10.1038/s41598-018-31171-5

**Published:** 2018-08-29

**Authors:** Elsa Seixas, Cristina Escrevente, Miguel C. Seabra, Duarte C. Barral

**Affiliations:** 1 Centro de Estudos de Doenças Crónicas, NOVA Medical School|Faculdade de Ciências Médicas, Universidade NOVA de Lisboa, Lisboa, Portugal; 2 Instituto Gulbenkian de Ciência, Oeiras, Portugal

## Abstract

Phagocytosis of invading microorganisms by professional phagocytic cells has a central role in innate immunity. However, several microorganisms developed strategies to subvert this process. Previously, we reported that bacteria and protozoa modulate differently the expression of Rab GTPases. Moreover, our results suggested that this modulation can contribute to avoid phagocytosis. Here, we investigated the mechanism by which the malaria parasite *Plasmodium berghei* and the bacterium *Escherichia coli* subvert phagocytosis through the modulation of Rab14 or Rab9a expression, respectively. We first confirmed that the scavenger receptor CD36 and the Toll-like receptor (TLR) 4 are required for the phagocytosis of *P. berghei* and *E. coli*, respectively. Interestingly, we observed that Rab14 silencing leads to an increase in the surface expression of CD36 in macrophages, which can explain the increase in the phagocytosis of *P. berghei* we reported previously. Similar results were obtained for Rab9a and TLR4, *i.e*. Rab9a silencing causes an upregulation of TLR4 surface expression in macrophages. Furthermore, we found that the decrease in the internalization of CD36 and TLR4, upon Rab14 or Rab9a silencing, respectively, can explain the increase in the surface levels of these receptors. Thus, our studies provide evidence that the modulation of phagocytosis caused by changes in Rab expression is operated, at least partly through changes in the surface levels of phagocytic receptors.

## Introduction

The innate immune system is the first line of defense against invading microorganisms. Professional phagocytic cells such as macrophages, dendritic cells and monocytes play a crucial role in the innate immune response and host defense. Central to this response is the expression of pattern recognition receptors (PRRs), such as scavenger receptors, which bind a broad array of modified and foreign ligands^1^. CD36 is a scavenger receptor expressed by myeloid cells, platelets, endothelial cells, erythroid precursors and adipocytes^2^. This receptor has been implicated in the immune response to modified host ligands such as oxidized lipoproteins, as well as foreign antigens like the malarial surface variant antigen, *Plasmodium falciparum* erythrocyte membrane protein 1^3,4^. CD36 is expressed on the surface of monocytes and macrophages and *in vitro* studies have shown that it mediates the phagocytosis of non-opsonized malaria-infected erythrocytes^5^. CD36 has also been identified as a candidate receptor for lipoteichoic acid (LTA), a cell wall component of the Gram-positive bacterium *Staphylococcus aureus*^6^. However, the mechanism by which CD36 orchestrates the response to pathogens and their ligands has not been defined.

Microbes are complex and present a wide variety of structures detected by the immune system. Other PRRs like the Toll-like receptors (TLRs) are expressed by phagocytic cells and recognize different microbial molecules from bacteria, viruses, fungi and protozoa. Upon ligand binding, TLRs activate common signaling pathways that initiate innate immune responses^7^. For instance, TLR4 mediates the signaling response to bacterial lipopolysaccharide (LPS)^8^. Moreover, TLR4 has been shown to be involved in the phagocytosis of *Escherichia coli*^9,10^. However, the mechanisms involved in the response to pathogens by TLRs remain poorly understood.

Phagocytosis is a complex process involving receptor binding, uptake and phagosome maturation^11,12^. Mature phagosomes fuse with lysosomes, forming a digestive organelle called phagolysosome that ultimately leads to the killing and digestion of the engulfed microbe(s)^13^. However, some pathogens can subvert this process or escape to the cytosol, avoiding digestion^14^. Rab small GTPases play important roles in phagosome maturation and phagolysosomal biogenesis. Indeed, host cell Rabs are critical for intracellular trafficking processes following phagocytosis^15,16^.

We previously reported that different pathogens modulate differently the expression of Rab proteins and that interfering with the expression of specific Rabs affects the phagocytosis of the pathogens^17^. Indeed, infection with *P*. *berghei* increases the expression of Rab14. Moreover, overexpression of Rab14 impairs phagocytosis of the parasites, whereas Rab14 silencing enhances it. Similar results were obtained with *E. coli* and Rab9a, *i.e*. infection with *E. coli* increases the expression of Rab9a and Rab9a overexpression impairs the phagocytosis of *E*. *coli*, whereas Rab9a silencing has the opposite effect. Thus, these results suggest that *P*. *berghei* and *E*. *coli* modulate the expression of Rab proteins to their own advantage.

Here, we investigated the relationship between the surface expression and endocytosis of phagocytic receptors and the expression of Rab GTPases. We also assessed how the modulation of the surface expression of these receptors impacts on pathogen phagocytosis by macrophages. We confirmed that CD36 is a receptor involved in the phagocytosis of the malaria parasite *P*. *berghei* and found that when Rab14 is silenced the levels of surface CD36 increase, which can explain the increase in phagocytosis we have observed previously. Similar results were obtained with TLR4 and *E*. *coli*. We also confirmed that TLR4 is a receptor involved in the phagocytosis of *E*. *coli* and its surface levels increase in macrophages silenced for Rab9a, which can explain the increase in phagocytosis we have reported. Furthermore, our studies indicate that the increase in the surface levels of CD36 and TLR4 is due to a decrease in their internalization. Thus, our studies suggest that the changes in phagocytosis caused by the modulation of Rab14 and Rab9a expression are caused, at least in part by changes in the surface levels of CD36 and TLR4, respectively.

## Materials and Methods

### Mice and parasites

Balb/c and C57BL/6 mice were bred at the specific pathogen-free (SPF) animal facility from *Instituto Gulbenkian de Ciência*. All procedures involving laboratory mice were in accordance with national (*Portaria* 1005/92, *Decreto-Lei* 113-2013) and European (Directive 2010/63/EU) regulations on animal experimentation and were approved by the *Instituto Gulbenkian de Ciência* and *Direcção Geral de Alimentação e Veterinária* ethics committees. One hundred thousand *Plasmodium berghei* ANKA-RFP-infected erythrocytes were injected intraperitoneally (i.p.) and parasitemia was measured by flow cytometry.

### Synchronization of parasites

*P*. *berghei*-infected erythrocytes were cultured *in vitro* to generate synchronized mature schizonts. Mice were bled 6 days after infection, red blood cells were resuspended in RPMI medium containing Fetal Bovine Serum (FBS), gassed with a mixture of 10% CO_2_, 5% CO_2_ and 85% N_2_ and incubated for 18 to 20 hours at 37 °C. Schizonts were enriched (>90%) using magnetically-activated cell sorting (MACS) as described previously^18^.

### Bacterial strains

*E*. *coli* M61655 K12 strain harboring a plasmid encoding YFP (*E*. *coli*-YFP) was inoculated in Luria Bertani broth with streptomycin and incubated overnight at 37 °C with vigorous shaking. The number of bacteria was calculated by flow cytometry using 2 μm beads.

### Macrophage differentiation

Bone marrow cells were isolated from mice femurs and tibias and differentiated *in vitro* for 7 to 8 days in Iscove’s medium supplemented with 10% FBS, 0.5 mM sodium pyruvate, 100 units/mL of penicillin, 100 mg/mL strepromycin, 5 × 10^−5^ M 2-mercaptoethanol and 30% L929-cell conditioned medium. The purity of the cell population was in all experiments greater than 90%, as determined by staining with anti-CD11b and F4/80 antibodies (eBiosciences).

### Macrophage and pathogen cultures

Primary macrophages at a concentration of 5 × 10^5^ cells per well were plated in 24-well plates and incubated with *Plasmodium*-infected erythrocytes or bacteria at a ratio of 30:1 (parasite:macrophage). After 15 minutes, cultures were washed with medium to eliminate extracellular pathogens and further incubated for 5, 15, 30 minutes or 1 hour.

### Flow cytometry

Cells were incubated with Alexa 647-conjugated anti-CD11b (eBiosciences), anti-CD36 (BD Biosciences) or anti-TLR4 (Abcam) antibodies, diluted in PBS containing 2% FBS and 0.01% NaN_3_. For blocking experiments, anti-CD36 (Abcam) and anti-TLR4 (eBiosciences) antibodies were used at 10 μg/mL and 20 μg/mL, respectively. Alexa 647-conjugated anti-mouse (Life Technologies) secondary antibody was used with anti-CD36 and FITC-conjugated anti-mouse (BD Biosciences) secondary antibody was used with anti-TLR4 antibody. Data were acquired and analyzed on a FACSCalibur using Cell Quest software (Becton Dickinson).

### siRNA silencing and rescue of Rab expression

siGENOME SMARTpool siRNAs (Dharmacon) specific for *Mus musculus* Rab14 or Rab9a were used to silence the respective Rabs. The list of siRNA sequences is shown in Table S1. Non-targeting siRNA pool (Dharmacon) was used as a negative control. Fourty pmol of siRNA were used to transfect primary macrophages according to the manufacturer’s instructions (Amaxa Biosystems). Bacterial or parasite infections were performed after 48 h of siRNA treatment. For the rescue experiments, after 48 h of silencing, the silenced Rabs were expressed. For that, primary macrophages where Rabs were silenced, were transfected with 2–5 μg plasmid DNA in nucleoporator buffer (Amaxa Biosystems) as with siRNA and cells were incubated for 6–8 h to allow for gene expression, prior to analysis by flow cytometry. The constructs used for expression of the GFP-Rabs are described elsewhere^17^.

### Internalization assays

Macrophages were plated at 5 × 10^5^ cells per well in 24-well plates, the anti-CD36 or anti-TLR4 antibody added and the cells incubated for 30 minutes on ice. After this time, cells were incubated for different time points at 37 °C to induce the internalization of the receptors. Anti-mouse FITC or Alexa 647 antibody was used to detect the receptors at the cell surface by flow cytometry.

### Statistical analysis

GraphPad Prism statistical analysis package (GraphPad Software, Inc., USA) was used to analyze experimental data. A p value < 0.05 was considered statistically significant using Student’s t-test for the analysis of statistical differences.

## Results

### CD36 and TLR4 are phagocytic receptors for *Plasmodium berghei* and *Escherichia coli*, respectively

Both CD36 and TLR4 are receptors that are expressed by macrophages and recognize pathogen-associated molecular patterns. It has been reported that CD36 is a receptor involved in the phagocytosis of malaria parasites^5,19^ and TLR4 in the phagocytosis of *E*. *coli*^9,10^. We have previously shown that Rab14 or Rab9a silencing leads to an increase in the phagocytosis of malaria parasites or *E. coli*, respectively. To determine if CD36 and TLR4 are involved in the increase in phagocytosis observed, we investigated the role of these receptors in the phagocytosis of *P*. *berghei* and *E*. *coli*, respectively.

**Figure 1:**
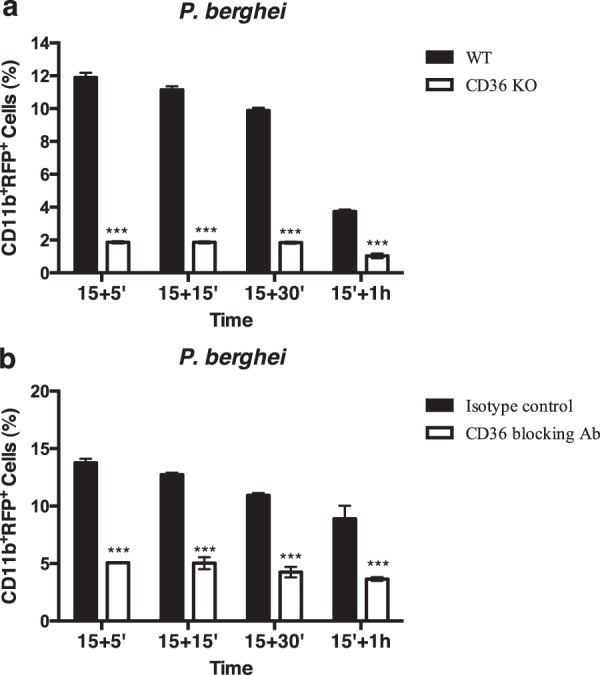
CD36 is required for the phagocytosis of *P. berghei* by macrophages. (**a**) Macrophages from CD36 knockout (KO) or wild-type (WT) mice were infected with RFP-*P*. *berghei*. (**b**) Macrophages were pre-treated with CD36 blocking antibody or with isotype control antibody and infected with RFP-*P*. *berghei*. Cells were incubated for 15 minutes, washed and chased for different time points. Cells were then analyzed by flow cytometry. Columns represent the percentage of positive cells for CD11b and RFP, analyzed after different times of incubation. Error bars indicate the standard error of the mean of four independent experiments. Statistical significance (***p < 0.001) refers to the difference between macrophages differentiated from KO mice and macrophages differentiated from WT mice (**a**) or to the difference between macrophages treated with blocking antibody and macrophages treated with isotype control antibody (**b**).

To study the role of CD36 in the phagocytosis of *P*. *berghei*, macrophages isolated from CD36^−/−^ mice were cultured in the presence of parasite-infected erythrocytes. We observed that the percentage of malaria parasites phagocytosed by CD36^−/−^ macrophages is markedly decreased, compared to wild-type macrophages (Fig. 1a). To confirm the requirement for the CD36 receptor in the phagocytosis of malaria parasites, we used macrophages pre-treated with a CD36 blocking antibody. As shown in Fig. 1b, treatment of macrophages with this antibody results in a significant inhibition in the uptake of *P*. *berghei*-infected erythrocytes. Therefore, these results further confirm the role of CD36 as a major receptor involved in the phagocytosis of infected erythrocytes by macrophages.

**Figure 2:**
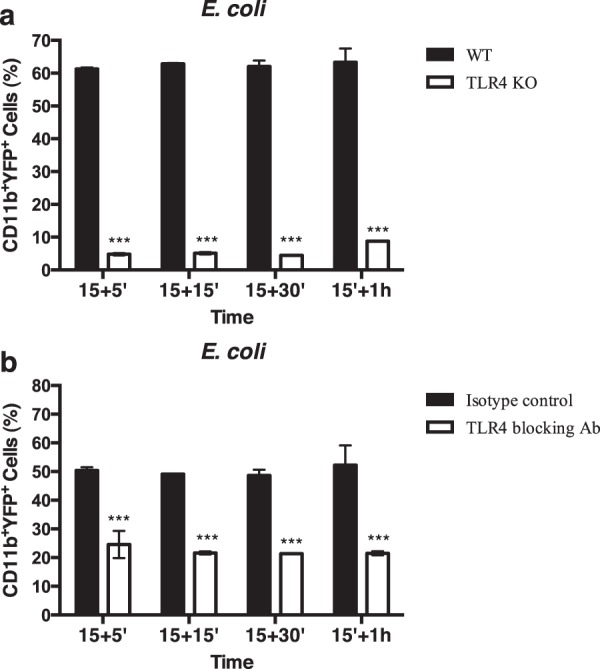
TLR4 is required for the phagocytosis of *E. coli* by macrophages. (**a**) Macrophages from TLR4 knockout (KO) or wild-type (WT) mice were infected with YFP-*E*. *coli*. (**b**) Macrophages were pre-treated with TLR blocking antibody or with isotype control antibody and infected with YFP-*E*. *coli*. Cells were incubated for 15 minutes, washed and chased for different time points. Cells were then analyzed by flow cytometry. Columns represent the percentage of positive cells for CD11b and YFP, analyzed after different times of incubation. Error bars indicate the standard error of the mean of four independent experiments. Statistical significance (***p < 0.001) refers to the difference between macrophages differentiated from KO mice and macrophages differentiated from WT mice (**a**) or to the difference between macrophages treated with blocking antibody and macrophages treated with isotype control antibody (**b**).

To determine the role of TLR4 in the phagocytosis of *E*. *coli*, macrophages from TLR4^−/−^ mice were cultured with bacteria for different time points. As displayed in Fig. 2a, macrophages from TLR4^−/−^ mice are greatly impaired in their capacity to phagocytose *E*. *coli*. Furthermore, we used a TLR4 blocking antibody to confirm the role of this receptor in the phagocytosis of *E*. *coli*. For this, macrophages were treated with TLR4 blocking antibody and the phagocytosis of *E*. *coli* determined by flow cytometry. We observed that TLR4 blocking results in a significant decrease in the phagocytosis of *E*. *coli* (Fig. 2b). Thus, these results further confirm the role of TLR4 in the phagocytosis of *E*. *coli*.

### CD36 or TLR4 blocking rescues the increase in phagocytosis caused by Rab14 or Rab9a silencing, respectively and re-expression of GFP-Rab14 or GFP-Rab9a rescues the increase in CD36 or TLR4 surface levels, respectively

**Figure 3:**
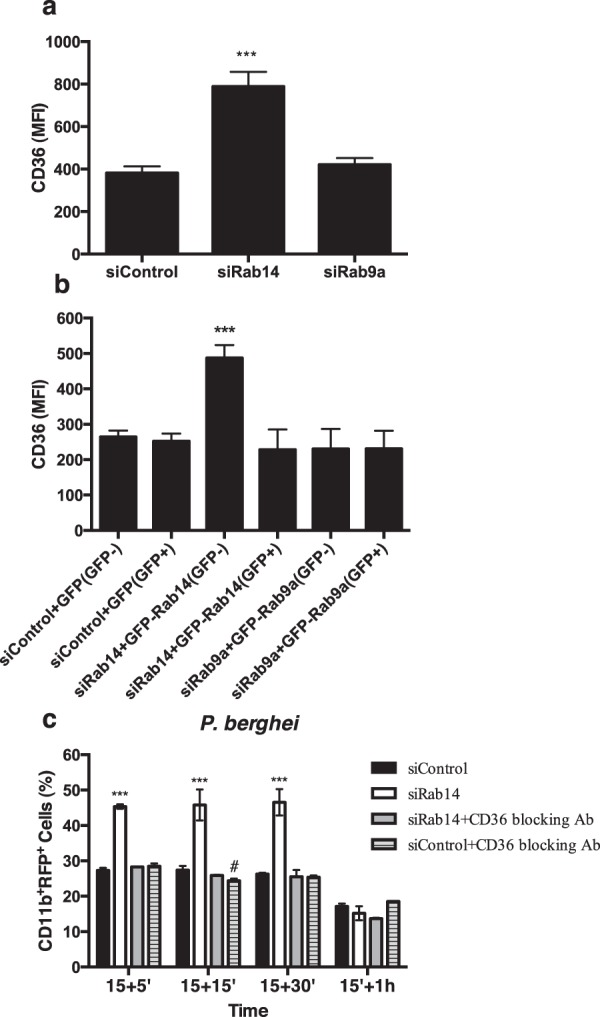
The increase in CD36 surface expression and phagocytosis of *P. berghei* caused by Rab14 silencing is rescued by the expression of GFP-Rab14 or blocking of CD36, respectively. (**a**) Macrophages were treated with siRNA for Rab14, Rab9a or siRNA control and the levels of CD36 analyzed by flow cytometry. Columns represent the mean fluorescence intensity (MFI) of CD36 in macrophages treated with the indicated siRNAs. Error bars indicate the standard error of the mean of four independent experiments. Statistical significance (***p < 0.001) refers to the difference between macrophages treated with siRNA for Rab14 and macrophages treated with siRNA control. (**b**) Macrophages were treated with siRNA for Rab14 or siRNA control, followed by expression of GFP-tagged Rab14 or Rab9a. Cell surface levels of CD36 of the GFP-negative and positive populations were analyzed by flow cytometry. Columns represent the mean fluorescence intensity (MFI) of CD36 in macrophages treated with the indicated siRNAs with or without expression of GFP-tagged Rabs. Error bars represent the standard error of the mean of two independent experiments. Statistical significance (***p < 0.001) refers to the difference between macrophages treated with siRNA for Rab14 and macrophages treated with siRNA control. (**c**) Macrophages were treated with siRNA control or siRNA for Rab14, incubated or not with CD36 blocking antibody and infected with RFP-*P*. *berghei*. Cells were incubated for 15 minutes, washed and chased for different time points. Cells were then analyzed by flow cytometry. Columns represent the percentage of positive cells for CD11b and RFP, analyzed after different times of incubation. Error bars indicate the standard error of the mean of four independent experiments. Statistical significance refers to the difference between macrophages treated with siRNA for Rab14 and macrophages treated with siRNA control (***p < 0.001) and to the difference between macrophages treated with siRNA control and macrophages treated with siRNA control and CD36 blocking antibody (^#^p < 0.05).

To investigate whether the increase in phagocytosis of malaria parasites or *E. coli* we have reported previously, results from an increase in the surface expression of CD36 or TLR4, respectively, we analyzed by flow cytometry the expression of these receptors on the cell surface upon silencing of Rab14 or Rab9a. Interestingly, we found that the silencing of Rab14 leads to an increase in the expression of CD36 on the surface of macrophages (Fig. 3a). Noteworthy, the silencing of Rab9a does not affect CD36 surface levels, showing the specificity of the increase in surface CD36 upon Rab14 silencing (Fig. 3a). Furthermore, we confirmed by RT-PCR that the silencing of Rab9a and Rab14 was efficient (Supplementary Fig. 1). To exclude off-target effects, we performed rescue experiments by re-expressing the silenced Rabs. We found that the expression of GFP-Rab14 leads to a reduction in CD36 surface expression to control values, in macrophages where this Rab was previously silenced (Fig. 3b). We confirmed by RT-PCR that the silencing and re-expression of Rab14 were efficient (Supplementary Fig. 2a). Together, these results suggest that the increase in the phagocytosis of *P*. *berghei* observed upon Rab14 silencing is a consequence of an increase in the surface expression of the phagocytic receptor CD36. To confirm that CD36 plays a role in the increase in phagocytosis observed upon Rab14 silencing, we cultured macrophages treated with siRNA for Rab14 with erythrocytes infected with malaria parasites in the presence of CD36 blocking antibody. We first confirmed that Rab14 silencing causes an increase in the phagocytosis of *P*. *berghei* by macrophages, compared to siRNA control (Fig. 3c). Strikingly, when CD36 blocking antibody was used in macrophages treated with siRNA for Rab14, the increase in the phagocytosis of infected erythrocytes was rescued, returning to control values (Fig. 3c). Therefore, these results strongly suggest that CD36 plays an essential role in the increase in the phagocytosis of *P*. *berghei*, observed upon Rab14 silencing.

**Figure 4:**
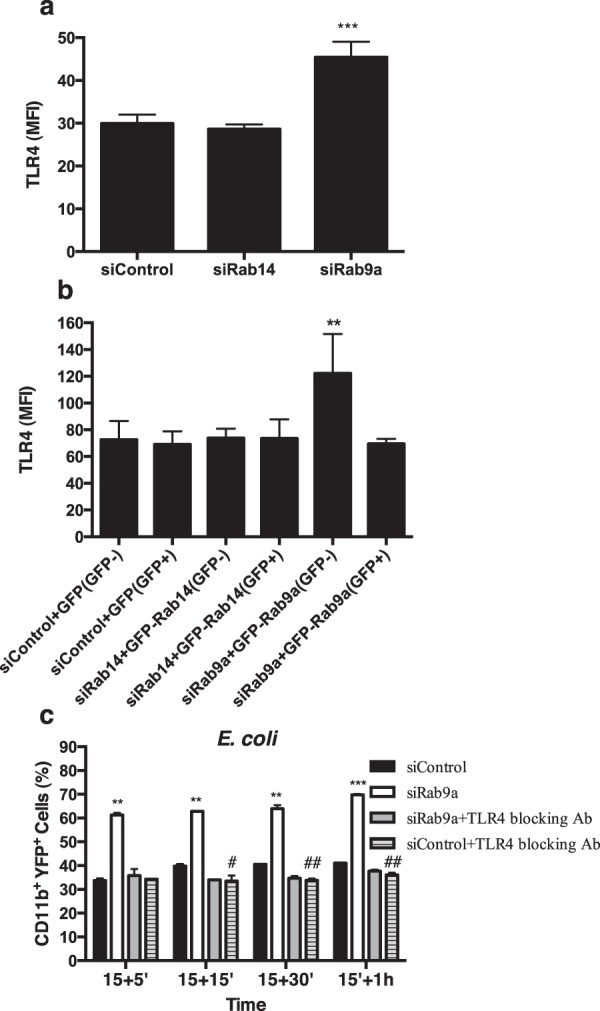
The increase in TLR4 surface expression and phagocytosis of *E. coli* caused by Rab9a silencing is rescued by the expression of GFP-Rab9a or blocking of TLR4, respectively. (**a**) Macrophages were treated with siRNA for Rab9a, Rab14 or siRNA control and the levels of TLR4 analyzed by flow cytometry. Columns represent the mean fluorescence intensity (MFI) of TLR4 in macrophages treated with the indicated siRNAs. Error bars indicate the standard error of the mean of four independent experiments. Statistical significance (***p < 0.001) refers to the difference between macrophages treated with siRNA for Rab9a and macrophages treated with siRNA control. (**b**) Macrophages were treated with siRNA for Rab9a or siRNA control, followed by expression of GFP-tagged Rab14 or Rab9a. Cell surface levels of TLR4 of the GFP-negative and positive populations were analyzed by flow cytometry. Columns represent the mean fluorescence intensity (MFI) of TLR4 in macrophages treated with the indicated siRNAs with or without expression of GFP-tagged Rabs. Error bars represent the standard error of the mean of two independent experiments. Statistical significance (**p < 0.01) refers to the difference between macrophages treated with siRNA for Rab9a and macrophages treated with siRNA control. (**c**) Macrophages were treated with siRNA control or siRNA for Rab9a, incubated or not with TLR4 blocking antibody and infected with YFP-*E. coli*. Cells were incubated for 15 minutes, washed and chased for different time points. Cells were then analyzed by flow cytometry. Columns represent the percentage of positive cells for CD11b and YFP, analyzed after different times of incubation. Error bars indicate the standard error of the mean of four independent experiments. Statistical significance refers to the difference between macrophages treated with siRNA for Rab9a and macrophages treated with siRNA control (**p < 0.01; ***p < 0.001) and to the difference between macrophages treated with siRNA control and macrophages treated with siRNA control and TLR4 blocking antibody (^#^p < 0.05; ^##^p < 0.01).

We also analyzed whether the silencing of Rab9a leads to an increase in the surface expression of TLR4 and confirmed that this is the case (Fig. 4a). Notably, the silencing of Rab14 does not affect TLR4 surface levels (Fig. 4a). To rule out off-target effects, we re-expressed the silenced Rabs and analyzed the rescue of the phenotype. We observed that the expression of GFP-Rab9a can completely rescue the increase in the surface expression of TLR4 caused by the silencing of this Rab protein (Fig. 4b). We also confirmed by RT-PCR that the silencing and re-expression of Rab9a were efficient (Supplementary Fig. 2b). Next, to confirm that TLR4 plays a role in the increase in phagocytosis observed upon Rab9a silencing, we infected macrophages treated with siRNA for Rab9a with *E. coli*, in the presence of TLR4 blocking antibody. We confirmed that Rab9a silencing leads to an increase in the phagocytosis of *E*. *coli* by macrophages, compared to siRNA control (Fig. 4c). Notably, when TLR4 blocking antibody was used in macrophages treated with siRNA for Rab9a, the increase in the phagocytosis of *E*. *coli* was not observed (Fig. 4c). Altogether, these results suggest that the increase in the phagocytosis of *E*. *coli* observed after Rab9a silencing is a consequence of the increase in TLR4 surface levels.

### Rab14 or Rab9a silencing leads to a decrease in the internalization of CD36 or TLR4, respectively

**Figure 5:**
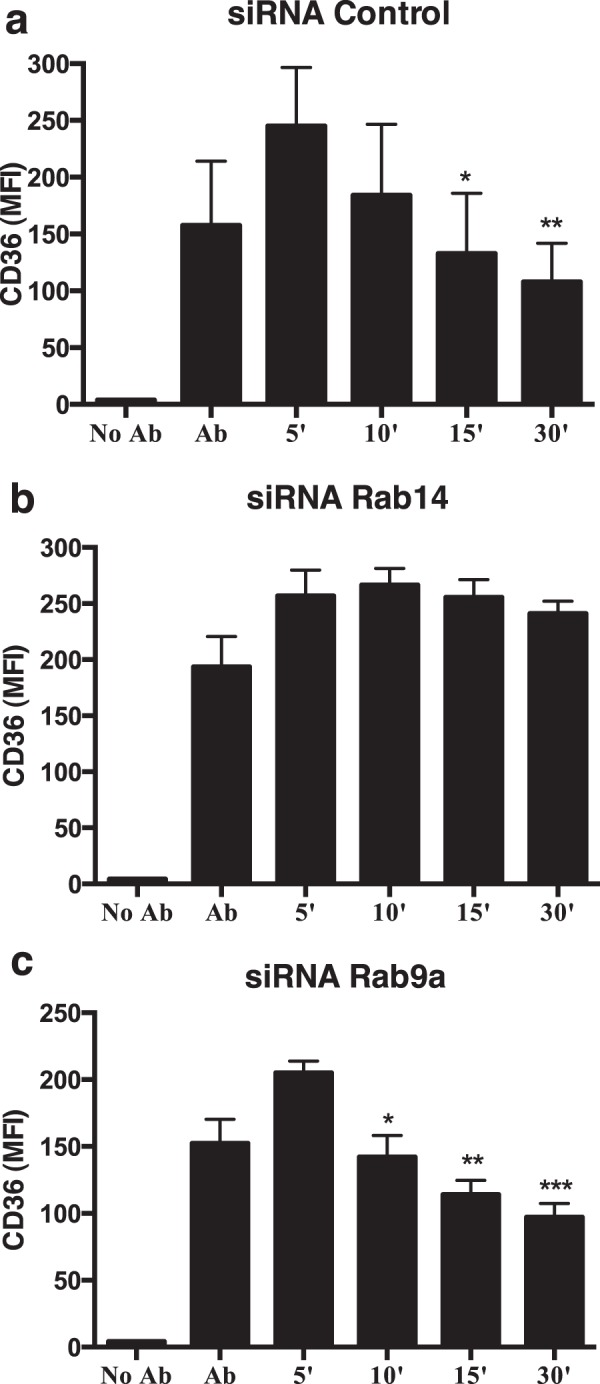
Rab14 silencing impairs the internalization of CD36. Macrophages were treated with (**a**) siRNA control, (**b**) siRNA for Rab14 or (**c**) siRNA for Rab9a and the surface levels of CD36 analyzed by flow cytometry. Columns represent the mean fluorescence intensity (MFI) of CD36 determined after different incubation times at 37 °C, using an anti-CD36 antibody. Error bars indicate the standard error of the mean of four independent experiments. Statistical significance (*p < 0.05; **p < 0.01; ***p < 0.001) refers to the difference between the 10, 15 or 30 minutes and 5 minutes incubation.

Our results indicate that the silencing of Rab14 or Rab9a leads to an increase in the surface expression of CD36 or TLR4, respectively. This increase in the surface expression of CD36 and TLR4 could result from a decrease in the internalization of these receptors from the cell surface. To test this hypothesis, we used antibody-induced cross-linking to drive CD36 and TLR4 internalization and analyzed the surface levels of these receptors by flow cytometry. When the cells were maintained at 4 °C, CD36 remained at the cell surface but when cells that had been incubated with anti-CD36 antibody in the cold were warmed up at 37 °C for different time points, we observed a decrease in the surface levels of CD36 (Fig. 5a). This confirmed that the receptor, bound to the antibody, was being internalized. However, when macrophages were treated with siRNA for Rab14, we observed a clear delay in the internalization of CD36 bound to the antibody (Fig. 5b). We also silenced another Rab protein, namely Rab9a, as a negative control and observed in this case a rate of internalization similar to the siRNA control (Fig. 5c). Therefore, these results suggest that the increase in the surface levels of CD36 upon Rab14 silencing results from a lower rate of receptor internalization.

**Figure 6:**
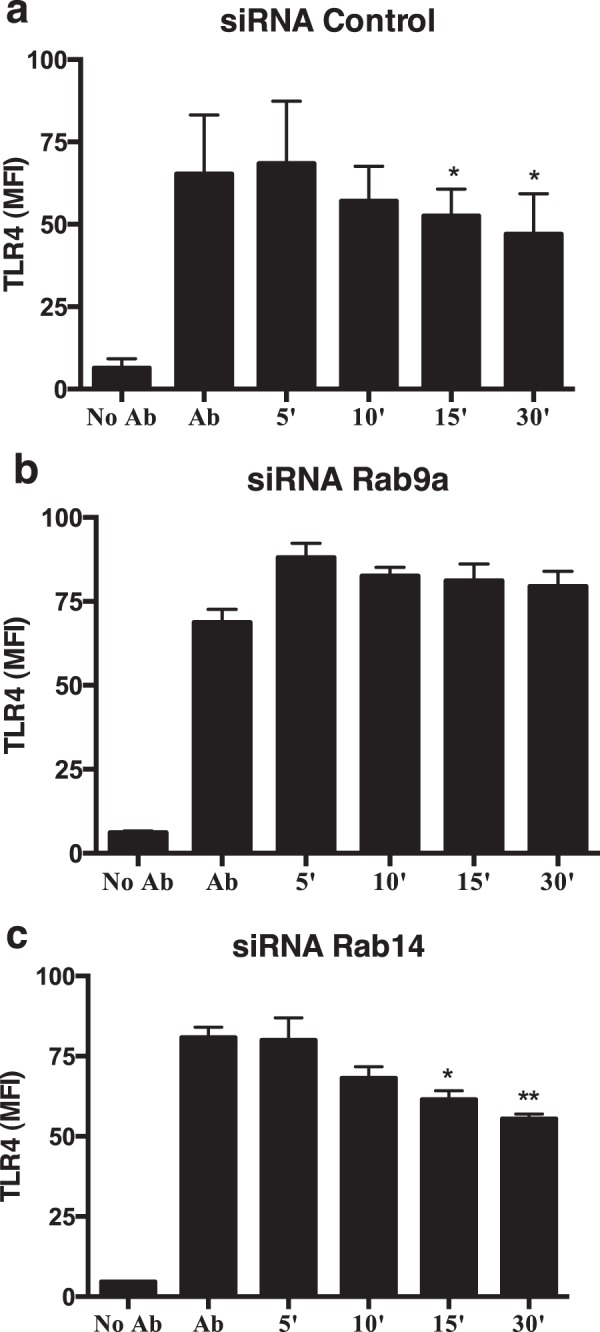
Rab9a silencing impairs the internalization of TLR4. Macrophages were treated with (**a**) siRNA control, (**b**) siRNA for Rab9a or (**c**) siRNA for Rab14 and the surface levels of TLR4 analyzed by flow cytometry. Columns represent the mean fluorescence intensity (MFI) of TLR4 determined after different incubation times at 37 °C, using an anti-TLR4 antibody. Error bars indicate the standard error of the mean of three independent experiments. Statistical significance (*p < 0.05; **p < 0.01) refers to the difference between the 10, 15 or 30 minutes and 5 minutes incubation.

The same strategy was used with Rab9a and TLR4. As with CD36, TLR4 surface levels decrease when cells are warmed up at 37 °C for different time points after treatment with siRNA control (Fig. 6a), indicating the internalization of the receptor bound to the antibody. However, when Rab9a was silenced, we observed only a small decrease in the surface levels of TLR4, indicating that in the absence of Rab9a the internalization of the receptor is impaired (Fig. 6b). Noteworthy, the silencing of Rab14 does not affect the internalization of TLR4 (Fig. 6c).

## Discussion

In this study, we present evidence that the increase in phagocytosis of *P. berghei* and *E. coli* caused by the silencing of Rab14 or Rab9a, respectively, is mediated, at least in part by the increase in the surface levels of major phagocytic receptors for these microorganisms, namely CD36 and TLR4. Moreover, the increase in the surface levels can be explained by a decrease in the internalization of the receptors, when Rab14 or Rab9a are silenced.

We first confirmed previous reports showing that CD36 acts as a phagocytic receptor in the internalization of malaria parasites^5,19^ and that TLR4 is a receptor involved in the phagocytosis of *E*. *coli* by macrophages^9,10^. Indeed, macrophages from CD36^−/−^ mice show impaired phagocytosis of *P. berghei*-infected red blood cells and similar results were obtained using a CD36 blocking antibody. We also observed an impairment in the phagocytosis of *E*. *coli* in macrophages derived from TLR4^−/−^ mice or that were treated with a TLR4 blocking antibody.

In a previous study, we showed that bacteria and protozoa can differentially modulate the expression of Rab GTPases, which are master regulators of membrane traffic^17^. Macrophages react specifically when infected with malaria parasites, upregulating the expression of several Rabs, including Rab14. On the other hand, after infection with *E. coli* we observed a distinct specific set of upregulated Rabs, including Rab9a^17^. We also showed that the silencing of Rab14 or Rab9a leads to an increase in *P*. *berghei* or *E*. *coli* phagocytosis, respectively. Since CD36 and TLR4 are involved in the phagocytosis of these pathogens, we went on to determine if the silencing of Rab14 or Rab9a affects the surface expression of these receptors. Indeed, this is the case, since the silencing of Rab14 leads to an increase in the surface expression of CD36 and the silencing of Rab9a to an increase in the surface expression of TLR4. Furthermore, treatment of macrophages silenced for Rab14 or Rab9a with blocking antibodies for CD36 or TLR4, respectively, inhibits the increase in phagocytosis caused by the silencing of the Rab proteins. This strongly suggests that the increase in phagocytosis observed upon silencing of Rab14 and Rab9a is due to the increase in the surface expression of CD36 and TLR4, respectively. Noteworthy, another study found that the silencing of Rab14 in cardiomyocytes has no effect on the surface expression of CD36^20^. The different results between this and our study could be explained by the difference in cell type, since macrophages are professional phagocytes and Rab14 function could be specialized in these cells due to its known role in phagocytosis.

To elucidate the mechanism by which the depletion of Rab14 or Rab9a leads to the increase in the surface expression of CD36 or TLR4, respectively, we postulated that these Rab proteins regulate the internalization of the receptors. Indeed, we found that Rab14 or Rab9a silencing impair the internalization of CD36 or TLR4, respectively, which can explain the increase in surface levels of these receptors and the consequent increase in the phagocytosis of *P. berghei* or *E*. *coli*.

CD36 stimulation by its ligands leads to the internalization of the ligand-receptor complex into endosomal compartments and the trafficking to the Golgi apparatus^21,22^. Interestingly, a role for Rab14 in trafficking of the glucose transporter GLUT-4, between early endosomes and the Golgi was described^23^. Moreover, Rab14 was shown to negatively regulate the surface expression of the UT-A1 urea transporter, through clathrin-mediated endocytosis^24^. Even though the internalization of CD36 seems to be clathrin-independent^21^, it is possible that Rab14 negatively regulates the surface levels of CD36 through a different internalization pathway. Similar to Rab14, Rab11a silencing increases CD36 cell surface levels^25^. Since Rab14 binds to Rab11-family interacting proteins (FIPs)^26^, which are known Rab11 effectors, the mechanism by which Rab14 controls the surface expression of CD36 could also be by antagonizing the function of Rab11, a master regulator of endocytic recycling. Indeed, Rab14 localizes to the endocytic recycling compartment, like Rab11^26^.

Rab9 is a small GTPase that localizes to the *trans*-Golgi Network (TGN) and late endosomes and has been reported to regulate membrane trafficking between these compartments^27^. Moreover, Rab9a is recruited at the time of Rab5-to-Rab7 conversion during endosome maturation^28^. Therefore, Rab9 might regulate the degradation of TLR4 in lysosomes and the impairment of this process could lead to an increase of the endocytic recycling of the receptor and consequently an increase in its surface expression^27^. Interestingly, the silencing of Rab7b, another regulator of late endocytic trafficking, leads to an increase in the surface expression of TLR4^29^.

In conclusion, our results show that the increase in phagocytosis of malaria parasites or *E. coli*, observed after Rab14 or Rab9a silencing, respectively, is due to an increase in the surface expression of the phagocytic receptors involved in the process. By interfering with the expression of specific Rabs, pathogens alter the surface expression of phagocytic receptors in order to avoid phagocytosis and intracellular killing. Hence, this work advances our understanding of the complex host-pathogen interplay and reinforces the importance of the innate immune system in the response to pathogens, in which the internalization of microbes by phagocytosis is a key event. Importantly, the understanding of this process is essential for the development of new therapeutic approaches to fight intracellular pathogens.

## Electronic supplementary material

Supplementary Information
