# Supplementary material for: Rab GTPase regulation of bacteria and protozoa phagocytosis occurs through the modulation of phagocytic receptor surface expression
Source: Sci Rep. 2018 Aug 29;8:12998. doi: 10.1038/s41598-018-31171-5 (PMC6115379; doi:10.1038/s41598-018-31171-5)
Supplement: Supplementary file 1 — Supplementary Information [file 41598_2018_31171_MOESM1_ESM.pdf]

Rab GTPase regulation of bacteria and protozoa phagocytosis occurs through the modulation of phagocytic receptor surface expression

Elsa Seixas, Cristina Escrevente, Miguel C. Seabra, Duarte C. Barral

Supplementary Information

Materials and Methods

Real-time Quantitative PCR

Total RNA was extracted using the RNeasy Mini Kit (Qiagen). 500 ng of total RNA was reverse-transcribed using SuperScript II Reverse Transcriptase (Invitrogen, CA) and random hexamer primers (Invitrogen). Reactions were incubated at 65ºC for 5 min, then at 25ºC for 10 min, followed by 42ºC for 50 min and finally at 70ºC for 15 min. Real-time quantitative PCR (RTqPCR) was performed in ABI Prism 7900HT system using ABI Power SYBR Green PCR Master Mix. The list of RT-qPCR primers used is shown in Table S2. The messenger RNA (mRNA) levels were normalized against Glyceraldehyde-3-phosphate dehydrogenase (GADPH).

Supplementary Figure 1 – Efficiency of Rab14 and Rab9a silencing after siRNA treatment. Macrophages were treated with (**a**) siRNA for Rab14, (**b**) siRNA for Rab9a, siRNA control or not treated (medium). Columns represent the relative quantification of the mRNA levels of each Rab, normalized to the mRNA levels of the housekeeping gene *GAPDH*. Error bars indicate the standard error of the mean of five independent experiments. Statistical significance (***p<0.001) refers to the difference between macrophages treated with siRNA for Rab14 or Rab9a and siRNA control.

Supplementary Figure 2 – Efficiency of Rab14 and Rab9a silencing and expression. Macrophages were treated with: (**a**) siRNA for Rab14, followed by expression of GFP-Rab14, (**b**) siRNA for Rab9a, followed by expression of GFP-Rab9a, or (**c**) siRNA control, followed by expression of GFP. Columns represent the relative quantification of the mRNA levels of each Rab, normalized to the mRNA levels of the housekeeping gene *GAPDH*. Error bars represent the standard error of the mean of two independent experiments. Statistical significance (***p<0.001) refers to the difference between macrophages treated with siRNA for Rab14 or Rab9a and siRNA control.

Suppl. Fig.1

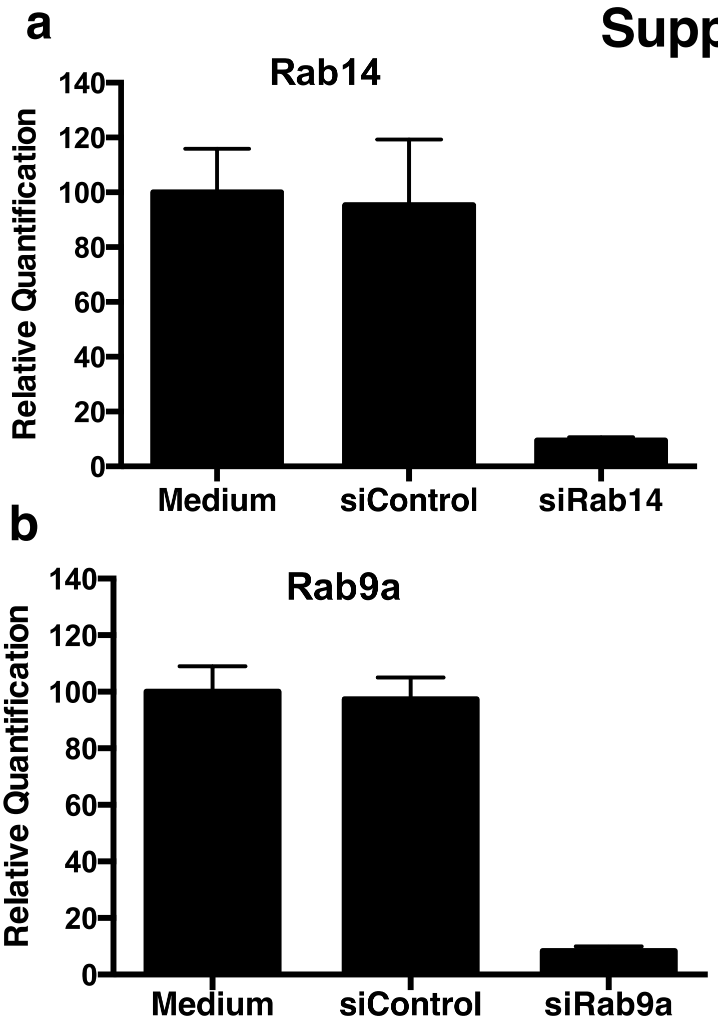
a
Rab14
Relative Quantification
140
120
100
80
60
40
20
0
Medium
siControl
siRab14
b
Rab9a
Relative Quantification
140
120
100
80
60
40
20
0
Medium
siControl
siRab9a

Suppl. Fig.2

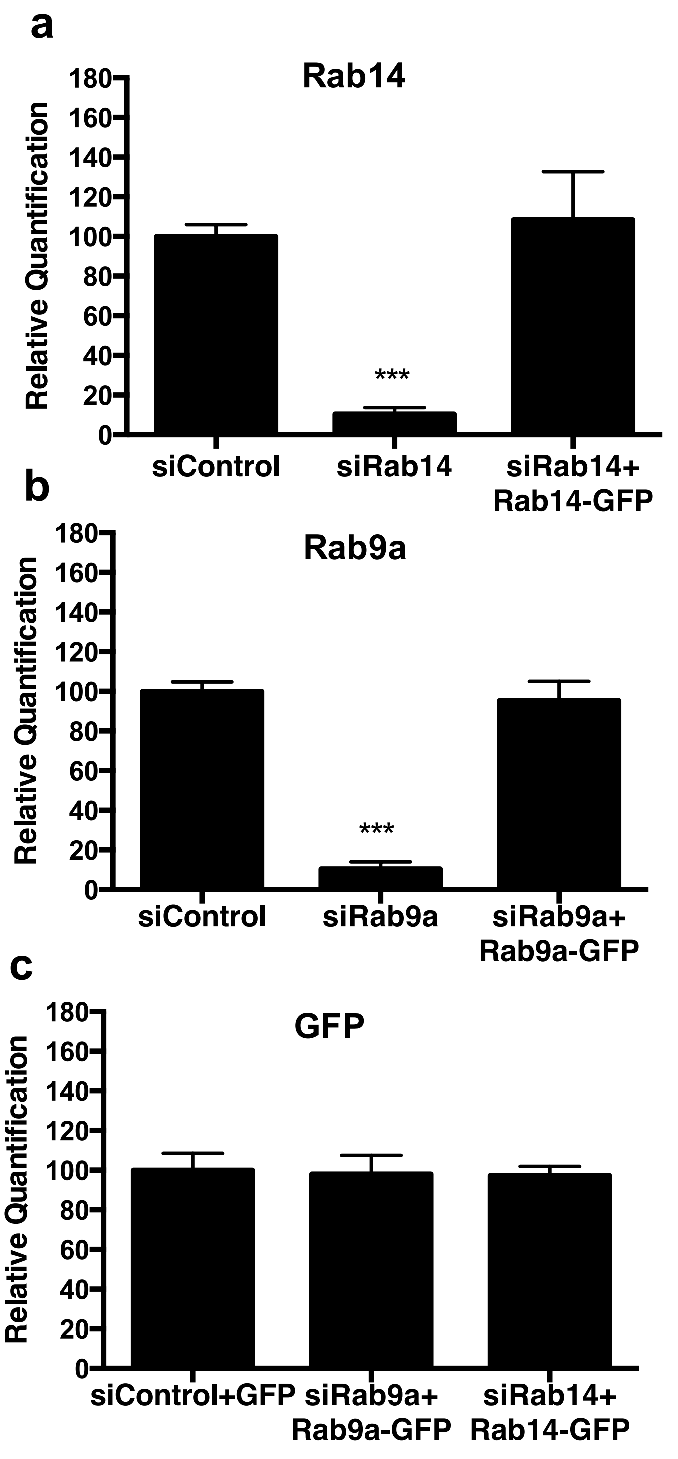
a
Rab14
Relative Quantification
180
160
140
120
100
80
60
40
20
0
***
siControl
siRab14
siRab14+
Rab14-GFP
b
Rab9a
Relative Quantification
180
160
140
120
100
80
60
40
20
0
***
siControl
siRab9a
siRab9a+
Rab9a-GFP
c
GFP
Relative Quantification
180
160
140
120
100
80
60
40
20
0
siControl+GFP
siRab9a+
Rab9a-GFP
siRab14+
Rab14-GFP
